# Substituting sugar confectionery with fruit and healthy snacks at checkout – a win-win strategy for consumers and food stores? a study on consumer attitudes and sales effects of a healthy supermarket intervention

**DOI:** 10.1186/s12889-016-3849-4

**Published:** 2016-11-22

**Authors:** Lise L. Winkler, Ulla Christensen, Charlotte Glümer, Paul Bloch, Bent E. Mikkelsen, Brian Wansink, Ulla Toft

**Affiliations:** 1 Research Centre for Prevention and Health, Centre for Health, Capital Region of Denmark, Rigshospitalet-Glostrup, Ndr. Ringvej 57, Building 84/85, 2600 Glostrup, Denmark; 2 Section of Social Medicine, Department of Public Health, University of Copenhagen, Copenhagen, Denmark; 3 Steno Health Promotion Research, Steno Diabetes Center, Niels Steensens Vej 8, 2820 Gentofte, Denmark; 4 Department of Clinical Medicine, Aalborg University, Frederikskaj 10, Building B, B2,, 2450 Copenhagen, SV Denmark; 5 Marketing in the Department of Applied Economics, Management at Cornell University, 114 Warren Hall, Ithaca, NY 14853 USA

**Keywords:** Food stores, Food environment, Checkout aisle, Environmental intervention, In-store marketing, Consumer research, Nutrition, Participatory approach, Field study

## Abstract

**Background:**

The widespread use of in-store marketing strategies to induce unhealthy impulsive purchases has implications for shopping experience, food choice and possibly adverse health outcomes. The aim of this study was to examine consumer attitudes and evaluate sales effects of a healthy checkout supermarket intervention. The study was part of Project Sundhed & Lokalsamfund (Project SoL); a Danish participatory community-based health promotion intervention.

**Methods:**

Consumer attitudes towards unhealthy snack exposure in supermarkets were examined in a qualitative pre-intervention study (29 short in-store interviews, 11 semi-structured interviews and three focus group interviews). Findings were presented to food retailers and informed the decision to test a healthy checkout intervention. Sugar confectionery at one checkout counter was substituted with fruit and healthy snacking items in four stores for 4 weeks. The intervention was evaluated by 48 short exit interviews on consumer perceptions of the intervention and by linear mixed model analyses of supermarket sales data from the intervention area and a matched control area.

**Results:**

The qualitative pre-intervention study identified consumer concern and annoyance with placement and promotion of unhealthy snacks in local stores. Store managers were willing to respond to local consumer concern and a healthy checkout intervention was therefore implemented. Exit interviews found positive attitudes towards the intervention, while intervention awareness was modest. Most participants believed that the intervention could help other consumers make healthier choices, while fewer expected to be influenced by the intervention themselves. Statistical analyses suggested an intervention effect on sales of carrot snack packs when compared with sales before the intervention in Bornholm control stores (*P* < 0.05). No significant intervention effect on sales of other intervention items or sugar confectionery was found.

**Conclusions:**

The present study finds that the healthy checkout intervention was positively evaluated by consumers and provided a ‘responsible’ branding opportunity for supermarkets, thus representing a win-win strategy for store managers and consumers in the short term. However, the intervention was too modest to draw conclusions on long-term sales and health implications of this initiative. More research is needed to assess whether retailer-researcher collaborations on health promotion can be a winning strategy for public health.

## Background

Checkouts and queuing areas are frequently used for an extensive promotion of colorfully packaged confectionery and other low-nutrient, high-sugar, calorie-dense food items strategically placed within the sight and reach of children [1–3]. Marketing research has shown how point-of-purchase (POP) promotion such as placement of products influences purchase [4, 5]. Placing products in end-of-aisle displays [6], on shelves at eyelevel and increasing the number of product facings [7] influences sales. Moreover, studies in supermarkets have found that unhealthy snacking products (e.g., chips and sugar confectionery) and sugar-sweetened beverages are largely impossible for costumers to avoid as they take up more shelf space than fruit and vegetables and are more often placed in displays at checkout and other high-traffic areas [8–10].

The widespread use of POP marketing strategies to induce unhealthy impulsive purchases has received attention from both consumers and researchers. Studies find consumer support for political and corporate restrictions in the way unhealthy products are placed and marketed, especially when targeting children [11, 12]. Campaigns in United Kingdom (UK) and Australia launched by consumer organizations have urged supermarkets to remove unhealthy snacks from checkouts and queuing areas [13–15]. In the UK consumer appeals have been met by some food retailer initiatives to reduce unhealthy snacks at checkout, but UK store audits have concluded that healthy checkouts remain an exception rather than a rule [13, 16]. Public health researchers are also attentive to the use of in-store marketing approaches and the links to obesity levels and diet-related diseases [17–19]. Some researchers point to the health-promoting potentials in using the same marketing strategies to make supermarket environments more supportive of healthy living [5, 20, 21]. Scientific literature examining feasibility, perceptions and sales effects of environmental interventions in real-life supermarket settings is sparse. Most of the existing knowledge from supermarket-based intervention studies is on the effect of using strategies aiming to increase nutritional knowledge or healthy product assortment rather than on strategies altering the accessibility and availability of both healthy and unhealthy food products [5, 22, 23]. To our knowledge no published papers specifically on interventions in real-life supermarket checkout areas exist. This study provides an exciting opportunity to advance our knowledge of using the supermarket as a setting for health promotion by describing the process and effect of a Danish supermarket-based healthy checkout intervention. The primary aim of this study was to examine consumer attitudes regarding roles and responsibilities of supermarkets in health promotion and to evaluate sales effects of a healthy checkout supermarket intervention.

### Project SoL and the supermarket intervention

The study was part of Project SoL (from the Danish Sundhed og Lokalsamfund / Health and Local Community), which was a community-based health promotion project taking place on the Danish island of Bornholm. The overall aim of Project SoL was to promote healthy shopping, eating and exercise behavior of families with 3–8 year old children. The intervention activities on Bornholm were carried out from summer 2012 to spring 2014 in three local communities with daycare centers, schools, local mass media and supermarkets as main intervention settings. Project interventions were developed and implemented by involving community stakeholders as the project was conceptually founded within an ecological and participatory approach; the so-called supersetting approach [24]. The overall project was evaluated using a quasi-experimental design comparing the intervention area, Bornholm, with a matched control area with similar socio-demographic characteristics, Odsherred.

Seven local supermarkets of different size and from three different food retail groups participated in the supermarket intervention on Bornholm: four stores from the Coop Group, two stores from the Dansk Supermarked Group and one store from Spar Denmark. The supermarkets contributed to the overall project aim by conducting in-store interventions using a mixture of structural, environmental and educational strategies and by engaging in community activities promoting healthy living.

The participatory approach implicated that the supermarket intervention was developed and evaluated drawing on the literature, formative consumer research and the experience and interests of the involved retailers. We integrated commercial marketing concepts and strategies in many in-store interventions drawing on the know-how of our supermarket partners and social marketing approaches [25, 26], for example by focusing on the products and promotional activities relevant to families with children. Qualitative data on food shopping habits of consumers and their perceptions of the health and promotion roles and responsibilities of participating supermarkets were collected at several occasions during the project to inform development of locally adapted health promotion activities and to enable feedback to store managers and researchers. Interviews were framed within an everyday-life perspective inspired by practice theory [27, 28]. Hence, we understand food shopping as a social practice; socially shared meanings and bodily routines embedded in material arrangements, which are constantly reproduced and modified by individual consumer ‘practitioners’.

**Fig. 1:**
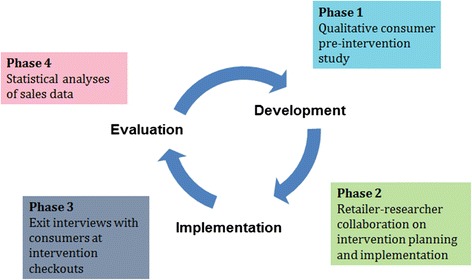
The four study phases

The present study included four of the participating stores all of which were part of the Coop Group (store 1-4, Table 1). The study was organized around four phases: 1) a pre-intervention study on consumer perceptions of in-store promotion of unhealthy snacks 2) development and implementation of a healthy checkout intervention 3) exit interviews on consumer intervention awareness and perceptions during the intervention 4) evaluation of intervention effects on sales (Fig. 1). Due to the interconnectedness and chronology of these study phases, the methods and results of each phase are reported separately before moving on to the next phase.

**Table 1:** Characteristics of the four Bornholm supermarkets participating in the Healthy Checkout intervention

Store	No. of staff members^a^ (approx.)	Size (approx.)
1	20	750 m^2^
2	20	850 m^2^
3	70	2000 m^2^
4	8	600 m^2^

^a^approximate number of staff members, number varies according to season and holidays. Numbers include full and part time staff and young workers

## Methods and Results

### Phase 1: qualitative pre-intervention consumer study

#### Phase 1: methods

The qualitative pre-intervention study was based on the formative consumer data collected to inform and evaluate supermarket intervention activities. Interview guides evolved around many aspects of everyday practices in relation to food provisioning and consumption. Thus, data were not originally collected to inform a healthy checkout intervention only, but the flexible interview guide included questions and vignettes on in-store inspiration and perceptions of the physical store environment. Unhealthy snack exposure was a naturally occurring discussion point introduced by both the interviewer and the participants across the interviews and we therefore found it relevant to look closer into this aspect of data. Data collection methods included 29 short in-store interviews (3–22 min), three focus group interviews with a total of nine participants (120–150 min) and 11 semi-structured telephone-based interviews (50–90 min). All participants lived in the three intervention communities and were recruited on store premises. To include as many participants from the target group-parents with smaller children-as possible, consumers perceived to be 25–45 years old and/or accompanied by a child were asked to participate more frequently than others. Most participants were recruited in two of the Coop stores (store 1 and 3) and in a discount store also participating in Project SoL as one of the original aims of the data collection was to inform and evaluate meal inspiration interventions taking place in the three stores. The number of participants in focus group interviews was low due to recruitment difficulties. Focus groups were therefore supplemented by telephone-based semi-structured interviews on the same themes. During the last semi-structured interviews, no essential new information or themes on food shopping practices were observed in the data indicating that data saturation was reached.

All interviews were audiotaped and transcribed verbatim. QSR Nvivo Software 10 was used to organize and analyze data. Following the steps of thematic analysis [29] all transcripts were read and re-read and repeated cycles of coding using both inductive and deductive approaches were undertaken. For this study, consumer accounts related specifically to promotion and placement of unhealthy snacks were identified across the heterogeneous data. Data were coded and collated into themes. LLW conducted the interviews and did the initial coding. Interpretation of data, theme identification and final theme selection was reviewed and discussed by multiple authors (LLW, UT and UC) throughout the analytical process to ensure a broad and critical reading and to validate findings. Extracts presented in this paper were translated from Danish to English.

#### Phase 1: results

Two dominant themes were identified and evolved around the challenges of snack exposure and the placement of responsibility for making healthy food choices.

#### Theme 1: tempting the ‘weak souls’

A common response regarding store exposure and promotion of unhealthy snacks and especially at the prominent checkout was that it may create controversies when families are shopping. Many consumers had witnessed encounters between parents and children concerning unhealthy snacks and some had similar negative shopping experiences with their own children. Some considered many of their fellow-consumers, especially children, to be unable to make rational decisions and to resist temptation when it came to snack exposure:

*“you overhear children asking for sweets and.. and I think it’s a really stupid idea to have candy and such things, which tempt children [at the checkout]”* (Woman 40’s, two children, baseline short in-store interview, store 3)

Moreover some participants admitted to be “weak souls” themselves when it came to unhealthy snack temptation. However, most participants perceived checkouts with unhealthy snacks to be an inappropriate store feature out of concern for other consumers.

#### Theme 2: ambivalent perceptions of store responsibility

As a consequence of the challenges imposed by unhealthy snack exposure at checkout, some participants would prefer if the stores removed unhealthy snacks altogether. However, most participants were more pragmatic and suggested that stores moved it to less prominent locations, placed it out of reach and sight of children and promoted healthier snack items at checkouts instead. Banning unhealthy snacks at checkouts was seen as a helpful gesture, but not as something that could be expected of stores. Most consumers found food choices, including choices about whether or not to buy unhealthy snacks, to be foremost an individual or parental responsibility:

“*I definitely think that the supermarkets have to run their business in order to make as much money as they can and then we’ll have to take the responsibility for our screaming kids”* (Woman 40’s, two children, semi-structured interview, store 1)

However, the responsibility of stores was also mentioned by some participants referring to the way price, placement and promotion was used strategically by the supermarkets. Moreover some participants observed contradictions between how the local supermarkets were presented in the media as active health promotion partners in Project SoL and how they lived up to this responsibility in practice. As an example, this participant had noted that some of the involved stores had supplemented the unhealthy products at checkout with healthy food products:

*“I think that they made a lot of it in the media considering what has happened in real life [..] I think it would have made them better off had they been consequent and then really removed all the candy instead of just placing three cucumbers at checkout”* (Man, 40’s, two children, focus group interview, store 3)

There were also consumers who addressed such discrepancy issues directly to staff members or to the local project coordinator (personal communication). Thus, to some consumers unhealthy snacks at checkout illustrated that the stores took neither their general health responsibility nor their participation in Project SoL seriously, whereas removing unhealthy snacks at checkout would be a signal of store willingness to help consumers make healthier choices.

### Phase 2: intervention development and implementation

#### Phase 2: methods – intervention development

Findings from phase 1 were presented to store managers and the regional sales manager from Coop at a joined planning meeting between the four participating stores and involved researchers. Although confectionery-free checkouts had been suggested as an intervention by researchers in the early phases of Project SoL while referring to findings and recommendations in marketing and public health literature and national consumer surveys, it was not until the store managers were confronted with the data on consumer perceptions from their own local communities that they agreed to take action. It followed from discussions at the meeting that community, social and business factors all played a role for retailers in arriving at the decision to test the effects of healthy checkouts. It was important for retailers to strengthen the shopping experience of especially community members with children. Competition within and across food retail groups influenced the decision as well. Store managers motivated and challenged each other and had a shared interest in gaining positive media coverage on this activity which they knew that the local media were eager to cover. The four Coop store managers and the regional sales manager concluded that a four week test of healthy checkouts was acceptable within the scope of their chain and company policies. The stores received no economic compensation from Project SoL in relation to participating in this and other project interventions.

The stores usually used their checkout to promote sugar confectionery (rather than for example chips, ice cream and sugary beverages). For the present intervention the stores agreed to substitute sugar confectionery with fruit and healthy snacks as suggested by consumers, but only at one of their checkout counters and only for a period of 4 weeks. Store 4 had one, store 1 and 2 had two and store 3 had a total of five checkout counters. The sugar confectionery category included chocolate bars, mints, liquorice, winegums and other items containing added sugar. It was up to each store to decide which healthy food and snack items to display instead, but the store managers coordinated their intervention product range to ensure a certain similarity in exposure, while maintaining the freedom to make local product adaptions. The idea was to provide healthy and convenient snacking options targeting especially parents shopping with children. Implementation was assessed and photographically documented by researchers or project assistants at weekly store audits during the intervention period.

#### Phase 2: results-implementation

The store managers wanted to get on with the intervention as soon as possible and the intervention was thus initiated 10 days after the planning meeting. It was implemented according to agreement in all four stores. One store had written down information (product name and barcode digits) on which products they displayed during the intervention. Unhealthy snacks were removed from intervention checkout counters (including adjacent island bin displays) and were replaced by a small selection of healthy snack products. The assortment included a mix of fresh fruit, dried fruit, dried fruit bars, unsalted nuts, and carrot snack packs and there was no difference between stores in how different intervention items were displayed.

The displays placed at the cash counter above the conveyor belt were used to promote healthy snacks in all stores. Additionally three of the stores used island bin displays and s-hooks at the checkout to make room for further healthy snack promotion.

In store 4 unhealthy snacks were removed at the entire aisle leading to the checkout and healthy snacks and non-food items were displayed instead. In store 1 unhealthy snacks were omitted from both checkouts and healthy snacks were placed on s-hooks located between the two checkouts, whereas healthy snacks were only placed at one of the checkout counters. In store 3 a “Healthy checkout” sign was hanged at the intervention checkout, but other than that no stores used signs, shelf labels or the project logo to promote and create awareness of the initiative.

While unhealthy snacks were less prevalent at checkout counters, unhealthy snack exposure continued to be high in other parts of the store. Store audits thus documented aisles with numerous shelves of unhealthy snacks adjacent to checkout areas. Furthermore large campaign displays promoted sugar confectionery in high-traffic end-of-aisle areas in the middle of store 3 and 4.

### Phase 3: Consumer exit interviews

#### Phase 3: Methods

To assess consumer awareness and perceptions of the healthy checkouts, short semi-structured exit interviews were made in two stores (store 1 and 3). The two stores were chosen for exit interviews as they had the largest number of consumers during the off-season time of the intervention and were located in separate parts of the island. Moreover, most qualitative formative project data were collected there.

The interviewer-administered semi-structured questionnaire consisted of six core questions including questions on awareness (“Have you noticed a new health initiative in store?”), attitudes (“What do think of such an initiative?” after telling them about the intervention if unaware about it) and perceived intervention influence on healthy choices (“does this initiative make healthy food shopping easier for you?” and “do you think this initiative make healthy food shopping easier for other customers?”). Consumers were approached as they were packing their groceries or when leaving an intervention checkout and asked if they would answer a few short questions about their shopping experience. Tourists were excluded. A total of 48 consumers participated: 23 from store 1 and 25 from store 3; of these 67% were women and the average age was 45.5 years. The interviews lasted 3–8 min. The interviewer had to be flexible and often left out some questions as many consumers were busy and on their way out of the store. Furthermore, seven of the participants (four from store 1 and three from store 3) were recruited after being in line at the checkout adjacent to the intervention checkout. Interviews were recorded on a digital voice recorder and subsequently fully transcribed. Six interviews were not audiotaped due to respondent unwillingness or technical errors and answers were written down instead. All interviews were conducted by the same interviewer (LLW). Quotes presented in this paper were translated from Danish to American English.

A thematic analysis [29] using a relatively descriptive approach was conducted. This analytical approach was chosen as we found the character and duration of our interviews to be insufficient for a more in-depth theoretical analysis. LLW conducted the analysis with input from several co-authors (UT, UC and PB). QSR Nvivo Software 10 was used to organize and code data. After the initial reading and re-reading of transcripts the data was categorized as related to awareness, attitudes or perceived helpfulness of intervention using exit interview questions as pre-defined categories. Second, responses within each category were coded to get an overview of the main tendencies. Third, a process of coding across categories was conducted as the initial coding within pre-defined categories was found to restrict some cross-cutting themes of the data. Finally, codes and themes were collated into three themes as presented below and illustrative quotes were selected.

### Phase 3: Results

#### Modest awareness of the checkout intervention

Awareness of the intervention was found to be modest as only few participants were able to mention the intervention when asked whether they had noticed a new health initiative in store during the last few weeks. Some participants pointed to a wider selection and increased exposure of fruit and vegetables and the occurrence of health-educational activities in the store. These were references to other on-going intervention activities taking place within Project SoL. Most of those participants who had noted the healthy checkout explained that they had observed the displayed fresh fruit and vegetables rather than the absence of sugar confectionery. Some also mentioned noticing the ‘Health checkout’ sign in store 3. A few consumers mentioned that they heard about the healthy checkout intervention in the local media. The majority was unaware of the change:

*“I didn’t notice, I didn’t look at the candy either. When you’re not bringing kids you don’t think about it”* (Woman 40’s, store 1)

Most participants simply explained that they “*did not notice*”, “*did not think about it*” or were preoccupied with other things.

#### Healthy checkouts: solidarity and store ethics

Attitudes towards the intervention were very positive as almost all participants used positive words when asked what they thought of the initiative, for example “*good idea*”, “*fine*” and “*super*”. These positive attitudes were often rooted in concern for children and their parents:

*“I think that it’s very good. I feel bad for parents when they stand here with their children and have to stand in such long queues waiting. Then it’s [candy] a bit difficult to avoid, when it’s right in people’s faces.”* (Woman 20’s, store 1)

This solidarity with other consumers who were shopping with children was observed among most participants and did not seem to differ across age and sex. Many participants had experienced their own children nagging for candy or had witnessed such episodes, which they found unpleasant for all involved.

Issues of store ethics and responsibility were also touched upon. Stores were complimented for taking a responsible stand by reducing temptations and participating in Project SoL: *“it shows morality in some way”* (Man, 50’s, store 1). At the same time it was acknowledged that the primary role of the supermarkets was to increase sales and profits. Hence, most participants maintained that food choices were first and foremost an individual or parental responsibility. Others saw the one healthy checkout as a positive, but relatively insignificant, step for promoting consumer health while pointing to the remaining abundance of sugar confectionery and other unhealthy snacks displayed throughout the store and near the checkout area:

Consumer: “*I actually think it is a bit immoral, you know, that it’s up here in this end [of the store]”*

Interviewer: *“So you think the store holds a responsibility for..”*

Consumer: (interrupts): *“Yes a little bit. They are allowed to sell candy, off course they are, but it doesn’t have to be at checkout”* (Woman 60’s, store 3)

Hence, to reduce exposure of sugar confectionery even more radically, for example by removing it from all checkouts and/or move it to more distant corners of the shop, was proposed by some participants.

#### Perceived helpful effects of intervention

While some participants expected the intervention to help them make healthier choices at checkout, a great majority of consumers believed that the intervention might help other consumers, especially those shopping with children.

Consumers, who did find the intervention helpful, indicated that the removal of unhealthy snacks was more important than the promotion of healthier alternatives, but both aspects of the intervention were considered important. The opportunity to avoid temptations within their-or their children’s-sight and reach and to grab a piece of fruit at checkout instead was considered appealing and helpful.

The many participants, who did not perceive the intervention to help them make healthier choices at checkout, explained that they were not easily tempted, did not buy a lot of candy in general, had no problems with nagging children due to consequent snacking agreements within the family or that they usually chose candy or fruit from other store locations than the checkout. Among both adult consumers and accompanying children some reservations regarding actually buying healthy snacks from the checkout were also expressed, referring both to low preferences for displayed snacks such as dried fig bars and skepticism on how healthy the displayed ‘healthier’ snacks actually were. However, not having small children (any longer) was the most frequent response:

“*It wouldn’t mean anything now, because now the children are older, so for me it wouldn’t mean a whole lot, no big difference. But I think that for families with children it would be really nice to remove it from the checkout”* (Woman 40’s, store 3)

Despite the modest intervention awareness and low expectations of behavioral effect on own shopping, the majority of participants were relatively confident that the intervention was helpful in reducing ‘pester power’-children nagging for unhealthy snacks-and impulsive unhealthy purchase among fellow consumers.

### Phase 4: sales data analyses

#### Phase 4: methods

Weekly sales data (revenue) for all products sold in 28 supermarkets from four chains owned by the Coop Group on Bornholm (intervention area) and in Odsherred (control area) in the period from August 19^th^ 2013 to December 1 ^th^ 2013 were included. The intervention group consisted of four supermarkets on Bornholm. There were two control-groups of supermarkets; the remaining 12 supermarkets on Bornholm (control group 1) and all 12 supermarkets in Odsherred (control group 2), thus giving a three level variable for intervention. The groups had a similar mix of supermarkets of different size and were considered comparable. A three level variable for period was also constructed to evaluate the intervention over time. The periods before and during the intervention each consisted of 4 weeks while the period after the intervention consisted of 7 weeks. The argument for defining the post-intervention period as longer than the pre-intervention period was that some of the stores maintained their healthy checkout intervention for up to 2 weeks after the termination of the official intervention period.

Healthy snack products displayed at checkout (store/week) during the intervention were identified based on documentation materials (pictures, notes and one store log book). For this study we included sales data on sugar confectionery (the entire category, including chocolate, winegums, liquorice and caramels) and the most frequently displayed intervention items: fresh fruit (overall), dried fruit (overall), dried fruit bars and carrot snack packs. Snack products only displayed at intervention checkout for at shorter period and/or in one or two intervention stores or with missing sales data were not included in the analyses, e.g., cherry tomatoes, cucumber snack packs and raisin snack packs. When defining outcome variables for overall sales of sugar confectionery, fresh fruit and dried fruit predefined category constructs from Coop were used, whereas variables of single-item snack products were defined by researchers based on text or barcode search in sales charts.

The intervention was evaluated using linear mixed models with the logarithm to sales data as the dependent variable. The fixed effects part of the model included the intervention variable and the period variable together with the interaction between the two variables. The random effects part of the model included a random effect for supermarket to allow for heterogeneity among supermarkets, and an autoregressive AR1 correlation structure to account for larger similarities of observations closer in time on the same supermarket. From this model estimates for the difference between the intervention period and the period just before and after the intervention were calculated for each intervention group. Tests to compare these estimates were calculated as well. Estimates and confidence intervals were back-transformed from logged data to original data for interpretive reasons.

Statistical analyses were performed using proc mixed in SAS statistical software v.9.4 (SAS Institute Inc., Cary, NC, USA).

#### Phase 4: results

We found no significant effect of the intervention on sales of neither confectionery (overall) nor sales of fruit (overall), dried fruit and dried fruit bars across time periods and when comparing sales in intervention stores to sales in both control-groups (data not shown). However data suggested a positive intervention effect on sales of carrot snack packs when comparing sales during and before intervention using Bornholm stores as controls (*P* < 0.05) (data not shown). Table 2 shows estimates from linear mixed models on sales of intervention snack items relative to reference periods within groups. Except for carrot snacks packs in one period in control stores all estimates were non-significant and with very wide confidence intervals. Nevertheless, the estimates can be used to indicate tendencies. Some estimates showed sales tendencies in the intended and hypothesized directions, e.g., sales of sugar confectionery decrease (by 4–7%), carrot snack packs increase (by 0.1–13%) and fruit bars increase (by 31–37%) in intervention stores when compared to periods 4 weeks before and 7 weeks after the intervention (Table 2). In comparison the sales of carrot snack packs and fruit bars decreased during the same period in both control groups, and for carrot snack packs sales decreases were significant. Nevertheless, data also showed decreased sales of fresh fruit and dried fruit during the intervention compared to before and after in the intervention group as well as in control groups (Table 2).

**Table 2:** Estimates from linear mixed models showing sales relative to reference period within store groups

Stores	Period	Sugar confectionery estimate[CI]	Fresh fruit estimate[CI]	Fruit bars estimate[CI]	Carrot snack pack estimate[CI]	Dried fruit estimate[CI]
Intervention stores^a^	Intervention period relative to 4 weeks before	0.93[0.80–1.06]	0.94[0.80–1.11]	1.31[0.78–2.20]	1.01[0.73–1.39]	0.80[0.58–1.11]
Intervention stores^a^	Intervention period relative to 7 weeks after	0.96[0.84–1.11]	0.92[0.78–1.09]	1.37[0.82–2.30]	1.13[0.82–1.56]	0.86[0.62–1.19]
Control 1^b^	Intervention period relative to 4 weeks before	0.95[0.87–1.03]	0.91[0.83–1.00]	1.03[0.75–1.42]	0.69[0.57–0.83]*	0.91[0.75–1.11]
Control 2^c^	Intervention period relative to 4 weeks before	0.95[0.88–1.04]	0.93[0.85–1.03]	0.85[0.62–1.17]	0.79[0.65–0.96]*	0.96[0.79–1.15]

^a^Intervention stores: the four intervention stores on Bornholm

^b^Control 1: Control group Bornholm

^c^Control 2: Control group Odsherred

*Bold data indicate significant estimates with values of *p* < 0.05

## Discussion

This paper reports on findings from four interconnected phases of a supermarket intervention in order to provide a rich and realistic description of a real-life setting intervention carried out by retailers and researchers in collaboration. First, an analysis of data from a qualitative pre-intervention study identified consumer annoyance with the exposure of unhealthy snacks within food stores. The analysis also showed that consumers were ambivalent about the extent to which stores are responsible for customer’s food choices. Second, it was described how the retailer-researcher dialogue was informed by these findings and that store managers were mainly motivated by the opportunity for local store profiling rather than by public health arguments when deciding to test healthy checkouts. The intervention was modest but implemented according to agreement. Third, the evaluation of the intervention by exit interviews showed that the healthy checkout intervention was positively received and considered helpful by almost all participants, but that most had not initially noticed the intervention. Lastly, we found no significant decrease in total sales of sugar confectionery, whereas data suggested an intervention effect on sales of one of the displayed healthy snack categories (carrot snack packs) when comparing sales during and before intervention using non-intervention stores on Bornholm as controls. However, this finding was primarily based on a decrease in carrot snack pack sales in the control groups. We also observed interesting but non-significant trends in the hypothesized directions of intervention item sales in the intervention period relative to reference periods within the intervention group. By using the four-phase processual approach and both qualitative and quantitative methods we have made an effort to demonstrate the dynamic process of a real-life setting intervention and provided insights and findings not usually reported from intervention studies.

We are not familiar with other published studies evaluating confectionery-free checkouts in a food store setting. Other supermarket-based intervention studies have enhanced the sales of healthy food products by using placement strategies [30–32], but have not intervened at checkout. However, Van Kleef et al. [33] tested the effects of altering the assortment structure and shelf arrangement of healthy and unhealthy snacks in an checkout display in a hospital staff canteen. They observed that displays consisting of 75% healthy snacks led to significantly higher sales of healthy snacks than displays consisting of 25% healthy snacks, while the intervention did not significantly impact sales of unhealthy snacks. These findings are in line with our results, although we only found significant increases in one category of displayed healthy products and were not able to measure the effect of assortment structure.

There are several possible explanations for why the healthy checkout intervention did not impact total sales of confectionery and only had modest effect on sales of healthy snacking products. One aspect to look at is how well we integrated the *marketing mix*-the four Ps: products, price, place and promotion-in our intervention [25].

Regarding *product* the healthy fruit and snack assortment displayed during the intervention was chosen with the target group in mind, for example by displaying fresh fruits as suggested by our participants. Furthermore, store managers and staff members were in charge of the specific implementation and product assortment to allow for some community and store differentiation. We lack appropriate data to explain why carrot snack packs was the most successful checkout item in our intervention. The Danish Coop Group has increased carrot snacks pack sales substantially during the 2000s [34] and health and convenience might be features making carrot snack packs successful. However, healthy assortments might not in general have the same ability to induce impulsive purchases as traditional calorie-dense snacks despite positive consumer attitudes towards this substitution. Studies find a discrepancy between healthy food choice intentions and actual behavior, for example Weijzen et al. (2009) documenting how a fourth of their study participants chose an unhealthy snack despite intentions to choose a healthy one [35].

We did not alter *price* on intervention items.

When it comes to *place* and *promotion,* covering aspects such as the physical location, promotional activities and communication, the central store placement of the intervention and the local media communication on the initiative were some of the successful features. The fact that the intervention only took place in Coop stores might also have played a role as one could hypothesize that Coop attract another customer group than discount stores, for example fewer from low-income groups and fewer young consumers. Our qualitative research indicated that the target group of Project SoL shopped regularly in all of their two or three local store options, but bought most of their groceries in a discount store, when this option was locally available.

We find other aspects related to place and promotion to be even more important. First, the duration of 4 weeks might have been too short to see behavioral effects. Second, the intervention included displays at and near *one* checkout counter, not all checkouts and not the entire queueing and checkout area. However, as already mentioned three of the intervention stores only had two checkouts and predominantly used one checkout at the off-season time of the intervention. Third, the use of signs and shelf labels to promote the intervention was modest. Fourth, and maybe most importantly, the confectionery that was removed from checkouts during the intervention comprised a modest quantity. Supermarkets devote more shelf space for unhealthy relative to healthy food products [8] and unhealthy products are extensively exposed on displays throughout stores [3, 36], reflecting that the confectionery removed from checkout in our study was just a small fraction of the confectionery exposed. Considering the competition between the promoted product and behaviors and other products and behaviors satisfying similar wants and needs, the healthy checkout intervention was up against serious in-store marketing odds. This helps to explain the low awareness and the fact that the presence of fruits and healthy snacks seemed to be noticed more than the absence of sugar confectionery.

We have examined target group practices and perceptions in accordance with participatory [24], practice-theoretical [27] and social marketing approaches [25, 26]. Hence, formative consumer research on food shopping practices and perceptions of the role and responsibilities of supermarkets in health promotion initiated this study in the first place. Still, our data did not allow us to make an in-depth analysis specifically on attitudes and practices related to snack exposure. Furthermore, consumer data from the involved local communities and stores were analyzed together. Hence, the design of the intervention could have been tailored more to meet specific local needs. Our evaluation design did not allow us to make sub-group comparisons to test whether the modest sales results “hided” an intervention effect among the family target group, and hence confirm or affirm consumer perceptions of who the intervention did or did not influence. Results indicate that the intervention was too modest to affect any group.

Taken together these discussion points point to a number of aspects which might have increased the intervention effect on sales, awareness and perceived influence:

- Target group pre-testing of specific intervention elements and products and more detailed data on shopping practices in different situations and store contexts in order to tailor the intervention more.
- More extensive exposure of the healthy checkouts using discounts and in-store promotional materials.
- Upscaling the intervention in terms of: reach (inclusion of all stores in the intervention communities), scope (inclusion of all store checkouts and the entire checkout area) and duration.

Our qualitative findings confirm and add to findings in other studies. Experimental studies find that changes in choice architecture can successfully promote certain behaviors within areas such as eating, but that individuals tend to deny being influenced by choice architecture while suggesting that other people are affected [4, 37]. This way of dissociating between oneself and others might also be related to a tendency to assess own health status and behavior in a favorable light [38, 39]. These findings are in agreement with our findings of the modest intervention awareness and the great belief in how the checkout intervention could be helpful to *other* consumers not least to those with children nagging for confectionery. However, qualitative studies examining the co-shopping practices of children and their parents [40, 41] indicate that child pestering might be an overrated phenomenon and that the relationship between the store environment and consumer practices is much more complex than our study and experimental studies are able to show. The related theme on ambivalent responsibility-participants stressing individual or parental responsibility for making healthy food choices on the one hand and the positive attitudes towards reducing store temptations on the other-was found across our qualitative data collected in different ways and across community and store settings. Furthermore, this ambivalent responsibility theme is in keeping with surveys showing broad support of statements stressing the individual responsibility for health while at the same time strongly supporting the idea of conducting health promoting interventions in public environments especially those targeting children [11, 42, 43]. This finding is also in line with studies identifying themes of individualization, self-control and self-responsibility in both consumer and food retailer discourses on healthy food choices [44–47].

While the scientific literature on the implications of impulsive marketing activities in real-life food stores is sparse, the food retail industry has valuable data and knowledge on these issues as they conduct marketing experiments on a daily basis. Examples of food retailers, that have banned unhealthy snacks from their checkouts, include Tesco and Sainsbury’s in the United Kingdom and Irma, a chain part of the Danish Coop Group [48, 49]. Results of such initiatives have not been made available for scientific scrutiny. However, it followed from press stories that the checkout policy in Irma has reduced overall sales of sugar confectionery and has not been compensated by increased healthy snack sales [50]. In contrast, a UK retailer from a company labelled as’proactive’ in an interview study stated that their healthy checkout chain policy did not damage neither short-term nor long-term profit; instead sales of confectionery multipacks from other prominent store locations increased [13]. Thus, the sales implications of confectionery-free checkout policies remain unclear.

Healthy checkouts might be a win-win strategy for consumers and food stores [51]. We showed that consumers see healthy checkouts as a positive signal of store willingness to act for the benefit of their costumer’s. For food stores, healthy checkouts is a way to brand themselves as responsible retailers and to strengthen customer loyalty without necessarily damaging overall profits. However, a permanent removal of unhealthy snacks at all checkouts involves an economic risk that many food retailers are not willing to take. Not surprisingly studies in food stores show that the top priority of food store managers and owners is making sales [13, 45, 52]. Hence, the uncertainty of fiscal implications of public health interventions challenges food store engagement [45, 52] and leads to the use of store owner monetary compensation and incentives as recruitment tools [53, 54]. While we acknowledge such barriers and motivations, our study shows that food store managers are willing to engage in community health interventions without economic compensation and that making profit is an important driver, but not the only one.

Healthy checkouts may contribute to public health gains, but the nutritional value of promoted ‘healthy’ snacks, substitution effects and possible unintended ‘compensatory’ in-store exposure of unhealthy snacks throughout the rest of the store must be taken into account. A related unintended public health implication of healthy checkouts and similar ad-hoc interventions might be that they increase sales of healthy snacks without necessarily decreasing sales of unhealthy snacks thereby increasing overall calorie consumption [19]. Due to such possible implications and the modest sales effect of the checkout intervention in our study, more studies examining the long-term sales and health implications of healthy checkouts and similar environmental changes are needed [51]. It will be interesting to see if proactive food retailers will lead the way in making store environments more supportive of healthy choices. The public health literature suggests that consumer pressure, public policy and ambitious public-private partnerships are needed to push for substantial and sustainable changes in the food retail environment [17, 18, 55].

### Strengths and limitations

Overall strengths of our study include the use of both objective measures of sales and subjective consumer accounts to examine the intervention from many perspectives. However, there are important limitations in our data collection as well. We collected only proxy data on individual behavior (sales data) and qualitative data on perceptions (exit interviews) rather than surveys with pre and post-tests, which means that the ability of our study to evaluate the intervention effect on consumer behavior and attitudes is limited. We consider it a great strength of this study that it was conducted in a real-life intervention setting and was developed and implemented in an iterative process with input from store managers, consumers and researchers. The latter might also be seen as a limitation as this participatory approach and iterative process did not allow us to have full control over intervention initiation, intervention implementation and the many others factors influencing sales in a supermarket. Moreover, we were working with small-size supermarkets with low product turnovers, which made changes in sales hard to detect statistically. Finally, Bornholm is an island with social and health indicators below average and an older population compared to the rest of Denmark [56] and it consists of small rural communities. This must be taken into account when considering the transferability of our findings. A similar intervention might be more successful in other areas, for example in urban areas with more families with small children.

## Conclusions

This paper reported findings from a study using qualitative as well as quantitative methods and a participatory approach to develop, implement and evaluate a healthy checkout intervention in a real-life supermarket setting. We conclude that the intervention was too modest to significantly affect sales. However, our study demonstrated the willingness of store managers to respond to local consumer wishes and the positive consumer feedback to this store initiative, thus potentially representing a win-win strategy for both store managers and consumers. More research with ambitious interventions regarding scope, reach and duration is needed to assess whether healthy checkouts can be a winning strategy for public health as well and whether our findings are transferable to other geographical and cultural contexts. Our study shows that food retailers and public health researchers can collaborate on community health matters, which is a good starting point.

Moving beyond the scope of healthy checkouts our study also reflects that the relationship between the socio-material contexts, such as store infrastructure, and the consumer practices embedded in such contexts is complex [27, 28]. Our study contributes to the sparse knowledge within a new and relevant research field crossing the borders between disciplines of public health, marketing, sociology and other behavioral sciences.

## Abbreviations

POP: point of purchase
Project SoL: Project Sundhed & Lokalsamfund (Health and Local Community)
UK: United Kingdom
